# LSR-YOLO: A High-Precision, Lightweight Model for Sheep Face Recognition on the Mobile End

**DOI:** 10.3390/ani13111824

**Published:** 2023-05-31

**Authors:** Xiwen Zhang, Chuanzhong Xuan, Jing Xue, Boyuan Chen, Yanhua Ma

**Affiliations:** 1 College of Mechanical and Electrical Engineering, Inner Mongolia Agricultural University, Hohhot 010018, China; zxw_imau@163.com (X.Z.); jingjingtuzi@126.com (J.X.); 15600899599@163.com (B.C.); yanhuama@126.com (Y.M.); 2 Inner Mongolia Engineering Research Center for Intelligent Facilities in Prataculture and Livestock Breeding, Hohhot 010018, China

**Keywords:** sheep identity recognition, deep learning, YOLOv5, lightweight improvement, mobile terminal recognition

## Abstract

**Simple Summary:**

The accurate identification of individual animals is an important step in precision breeding. However, the traditional identification methods have large defects. With the continuous development of computer vision and deep learning technologies, it is possible to establish accurate biological recognition models. In this study, we built a lightweight sheep face recognition model using convolutional neural networks (CNNs) to face the challenges in sheep identity recognition. The model not only achieved high accuracy in recognition tasks but also was friendly for edge devices. The research results indicate that the proposed recognition model can identify sheep accurately and can be further deployed on the mobile end.

**Abstract:**

The accurate identification of sheep is crucial for breeding, behavioral research, food quality tracking, and disease prevention on modern farms. As a result of the time-consuming, expensive, and unreliable problems of traditional sheep-identification methods, relevant studies have built sheep face recognition models to recognize sheep through facial images. However, the existing sheep face recognition models face problems such as high computational costs, large model sizes, and weak practicality. In response to the above issues, this study proposes a lightweight sheep face recognition model named LSR-YOLO. Specifically, the ShuffleNetv2 module and Ghost module were used to replace the feature extraction module in the backbone and neck of YOLOv5s to reduce floating-point operations per second (FLOPs) and parameters. In addition, the coordinated attention (CA) module was introduced into the backbone to suppress non-critical information and improve the feature extraction ability of the recognition model. We collected facial images of 63 small-tailed Han sheep to construct a sheep face dataset and further evaluate the proposed method. Compared to YOLOv5s, the FLOPs and parameters of LSR-YOLO decreased by 25.5% and 33.4%, respectively. LSR-YOLO achieved the best performance on the sheep face dataset, and the mAP@0.5 reached 97.8% when the model size was only 9.5 MB. The experimental results show that LSR-YOLO has significant advantages in recognition accuracy and model size. Finally, we integrated LSR-YOLO into mobile devices and further developed a recognition system to achieve real-time recognition. The results show that LSR-YOLO is an effective method for identifying sheep. The method has high recognition accuracy and fast recognition speed, which gives it a high application value in mobile recognition and welfare breeding.

## 1. Introduction

With the continuous development of precision agriculture, precise and intelligent breeding methods have been widely discussed. In modern farm management, it is necessary to collect different types of information on sheep, such as vaccination information and pregnancy status. Collecting different types of information can help farmers manage their farms, further develop effective management strategies, improve feeding methods, and reduce feeding costs [1,2]. Before collecting various information about individual sheep, it is necessary to determine their corresponding identities. Meanwhile, sheep identification can help prevent diseases and further promote sheep growth. In addition, the identification of individual sheep can lead to the traceability of meat product quality and further meet the needs of people for high-quality meat. Therefore, the automatic identification of individual sheep has become indispensable.

Traditional sheep recognition methods include paint marking, manual observation, and invasive equipment technology [3,4]. However, traditional methods have limitations. The manual observation method has low efficiency and accuracy and is not suitable for large-scale sheep flocks. The paint marking method requires frequent maintenance and cleaning. The use of radio frequency identification (RFID) tags can bring pressure on animals. In addition, tags are often damaged, lost, and easily disturbed in complex environments [5,6]. Considering that sheep are usually raised in groups, which makes it difficult and time-consuming to collect information about each sheep, it may be inconvenient for farmers to manage their farms by relying on traditional sheep recognition methods [7].

With the development of information technology, biological image recognition has received more and more attention and has become a promising trend in animal identification. Biological image recognition technology takes advantage of intelligent monitoring equipment and computer vision to obtain the stable biological features of sheep, including their DNA fingerprints, iris patterns, and facial images [8,9]. Among these methods, recognition methods based on iris patterns and DNA fingerprints face many challenges. Collecting clear and stable iris images is relatively difficult, and changes in brightness during the collection process can easily lead to acquisition failure [10,11]. The accuracy of identifying individual sheep through DNA fingerprints is high, but the recognition time is long, so real-time detection cannot be achieved. In contrast, sheep face recognition is a low-cost and efficient recognition method that is currently the mainstream research direction for sheep biological image recognition.

In recent years, scholars have used computer vision technology to recognize livestock faces, and various CNNs have been developed for the task of identification [12,13,14]. Song et al. [15] used an improved YOLOv3 model to recognize 20 adult Sunit sheep, and the mAP reached 97.2%. Although the model size of improved YOLOv3 has been reduced from the initial 235 MB to 61 MB to reduce computational costs, the recognition model still has large parameters, which is not conducive to deployment on mobile devices. In addition, the number of experimental sheep is relatively small, and it is difficult to identify comprehensive and detailed sheep face features in the constructed sheep face dataset. Billah et al. [16] collected 3278 photos of goats, including open-source images and manually captured facial images of 10 dairy goats, and used the YOLOv4 model for facial recognition, achieving a recognition accuracy of 96.4%. However, the model size of YOLOv4 is 244 MB, so it does not have advantages in terms of model size or recognition speed. Considering that YOLOv3 and YOLOv4 are versions before the YOLO series, although they have achieved high performance in sheep face recognition tasks, the models are relatively large in model size and are not suitable for practical applications of sheep face recognition. Hitelman et al. [17] used the ResNet50V2 model combined with the ArcFace loss function to train the facial images of 81 young Assaf sheep with an average recognition accuracy of 97%. However, the size of the ResNet50V2 model is about 98 MB, and the model parameters are too large, which is not conducive to deployment on mobile devices. Although CNNs have achieved good results in sheep face recognition, the sizes of the relevant sheep face recognition models are too large, the recognition times are long, and the costs of deployment to mobile devices are not considered. Deploying a sheep face recognition model on mobile devices meets the needs of practical applications. In practical applications, herders can collect, identify, and save information on sheep at any time, making it more convenient and efficient to collect various information about sheep and further improving the efficiency of farm management. In addition, compared to the upper computer control system, the cost of designing and developing a mobile recognition system is lower. To our knowledge, there is currently limited research on lightweight sheep face recognition models and mobile system design, and further evaluation and development are needed.

YOLO (You Only Look Once) is a high-performance recognition model [18,19,20]. YOLOv5 has attracted more and more attention with the development of the YOLO series of algorithms [21,22]. There are four versions of YOLOv5, of which the YOLOv5s model has obvious advantages in FLOPs and parameters. The model size of YOLOv5s is 14 MB, which shows the potential for its deployment on an intelligent mobile terminal. In this study, an improved lightweight model based on YOLOv5s was developed and named LSR-YOLO. Firstly, the lightweight ShuffleNetv2 module replaced the feature extraction module in the backbone of YOLOv5s, effectively reducing the model size and FLOPs. Through the comparison of several improved models, we found that the loss of mAP@0.5 was minimal when the Ghost module was introduced into the neck of YOLOv5s. For the C3 module in the neck of YOLOv5s, we integrated the Ghost module and further built a lightweight C3Ghost module to reduce the model size and FLOPs. Finally, the CA attention module was introduced in the backbone to enhance the feature extraction ability of recognition model. Extensive experiments showed that the LSR-YOLO achieves the desired performance compared to existing detection methods. The main contributions of this study are as follows:

(1) A novel, lightweight sheep face detection method called LSR-YOLO was proposed. The model size of LSR-YOLO is only 9.5 MB. Experiments showed that LSR-YOLO achieves a good balance in detection efficiency, model size, and detection accuracy.

(2) We deployed LSR-YOLO on the mobile end and further designed a mobile recognition system, which provides technical support for the development of sheep face recognition system on the the mobile end. 

The paper is organized as follows: in “Section 2,” we introduced the details of shooting sheep facial images in detail and the steps of constructing sheep face dataset. In addition, we introduced the model architecture and the details of the improved modules. In “Section 3,” we described the details of the comparison experiments and presented the detailed experimental results. In “Section 4,” we introduced the facial image acquisition device of sheep, the sheep face mobile recognition system, and future research directions. “Section 5” summarizes the research of this paper.

## 2. Materials and Methods

### 2.1. Self-Built Dataset

#### 2.1.1. Data Collection

**Figure 1:**
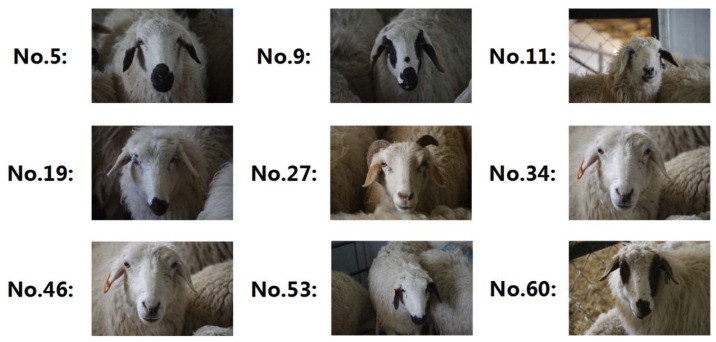
Randomly selected examples of experimental sheep.

The experiment of this study was conducted on a group of small-tailed Han sheep. The characteristic of small tailed Han sheep is that some of their faces have black or brown spots. The black and brown spots on their faces are mostly concentrated around their eyes, ears, cheeks, or mouth [23]. The sheep facial images used in this study were captured at the Tianjin Aoqun Animal Husbandry Co., Ltd. (Tianjin, China). The collection date was August 2020. In the experimental area, the experimental sheep were concentrated in the sheepfold. The sheep facial images were captured using a single lens reflex camera (Canon EOS 600D, Canon, Tokyo, Japan) and saved in JPG format with an image resolution of 2736 × 1824. To make the collected dataset more complex and suitable for international applications, diverse collection methods were adopted, including different lighting conditions, shooting angles, and shooting distances. The interval between capturing each facial image was greater than 10 s to prevent the collected images from being highly similar. A total of 63 experimental sheep were used in the study, and the age of the experimental sheep was controlled between one and two years old. A total 100 facial images were collected for each experimental sheep. The experimental sheep were sorted according to serial numbers from 1 to 63, which corresponded to their identity information. Figure 1 shows examples of randomly selected experimental sheep.

#### 2.1.2. Dataset Pre-Processing and Creation

**Figure 2:**
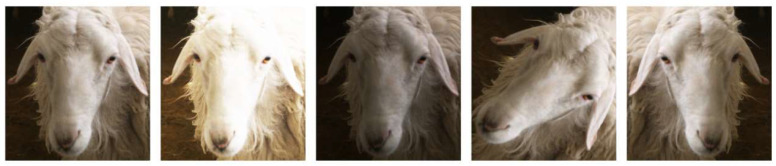
The corresponding operations, from left to right, are the original image, brightened image, darkened image, image randomly rotated, and image vertically flipped.

We performed data cleaning on the original collected sheep facial images and further removed blurry images with a manual inspection. A total of 6110 images were retained for file labeling after cleaning. Then, the sheep face dataset was extended using data enhancement methods. The specific operations were as follows: adjusting image brightness, random rotation of 45 degrees, and vertical flip. Samples of the data enhancement effects are shown in Figure 2. Using the above methods, 100 enhanced images were supplemented for each experimental sheep, which were used for model training.

Make Sense is a label-making tool that was used to mark the position of the sheep face and generate corresponding label files. As shown in Table 1, the sheep face dataset was randomly divided into a training set, testing set, and verification set with a ratio of 8-1-1.

### 2.2. LSR-YOLO Network Architecture Design

**Figure 3:**
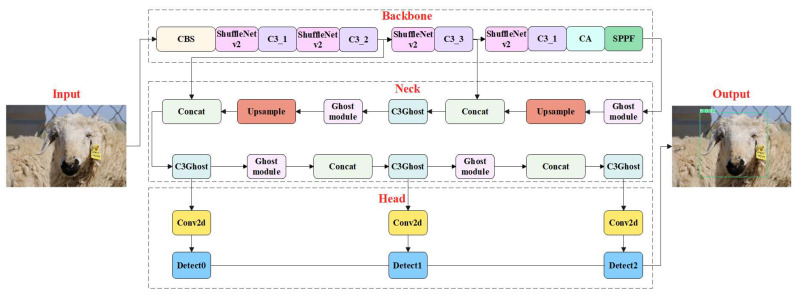
Schematic diagram of LSR-YOLO. LSR-YOLO mainly includes the backbone, neck, and head, with lightweight ShuffleNetv2 module (pink) and attention mechanism CA module (cyan) added to the backbone. In the neck, the model size and parameters were further reduced by introducing the Ghost module (light pink) and C3Ghost module (light cyan).

In this study, we embedded the CA module in front of the SPPF module to improve the model performance in recognition tasks. After the introduction of the CA module, the new feature map obtains the attention weight in the channel and spatial dimensions. In addition, we replaced the CBS module in the backbone with the ShuffleNetv2 module to reduce the parameters and model size. Furthermore, in the neck network of YOLOv5s, the C3 module and original convolution module were replaced with the C3Ghost module and Ghost module. By introducing C3Ghost, and Ghost modules into the improved model, the model size, parameters and FLOPs can be further reduced. The schematic diagram of LSR-YOLO is shown in Figure 3.

#### 2.2.1. Sheep Face Detection Module

YOLOv5 integrates a variety of optimization modules. YOLOv5 can be divided into four versions, including YOLOv5s, YOLOv5m, YOLOv5l, and YOLOv5x. Among these models, YOLOv5s has the smallest model size and the fastest detection speed [24]. Therefore, YOLOv5s was selected as the basic model in this study, and lightweight improvement was carried out on its basis. The YOLOv5s model used in this study was v6.1. Compared with the previous version, v5.0, the network structure of v6.1 is more streamlined [25].

YOLOv5s is composed of an input, backbone, neck, head, and output. Specifically, the input end includes adaptive picture scaling, mosaic data enhancement, and adaptive anchor box calculation. Adaptive picture scaling can uniformly reduce original images with different lengths and widths to a standard size, reducing them according to the length–width ratio of the original image and filling the reduced image with gray to ensure a consistent size of the input image. Mosaic data enhancement randomly selects four images for clipping and randomly arranges and stitches the cropped images, which can enrich the dataset and improve the training speed of the model by training four images at a time. Adaptive anchor box calculation uses K-means and genetic learning algorithms to analyze the dataset and further obtains preset anchor boxes suitable for identifying target boundary boxes [26]. 

The backbone network is composed of CBS, CSP1, and Spatial Pyramid Pooling Fast (SPPF) modules. CBS is the basic feature extraction module of YOLOv5s. The CBS module and residual structure modules constitute the CSP1 module. The CSP1 module contains two branches and can effectively retain the feature information of different branches through a Concat operation connection to extract more abundant feature information. Its residual structure can avoid the disappearance of gradients due to the deepening of the network. The SPPF module uses three 5 × 5 maximum pooling layers to effectively solve the problems of incomplete image cropping and shape distortion as well as to obtain more feature information by fusing more features of different resolutions. Compared with the SPP module, the SPPF module reduces the amount of computation while ensuring similar accuracy [27,28].

**Figure 4:**
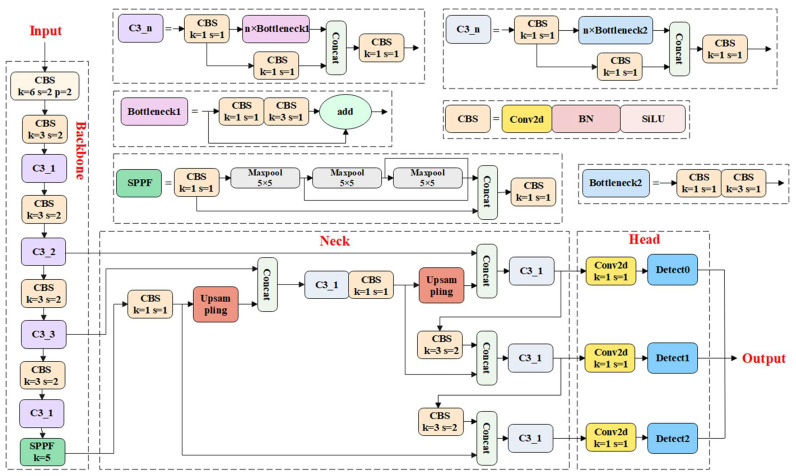
Architecture diagram of YOLOv5s. CBS (orange) is the basic component module of YOLOv5s, used to extract target features. In the backbone, detailed target features are extracted through the C3P1 module (purple), and finally feature fusion is performed through the SPPF module (green). The CSP2 module (light gray) is used in the neck to extract target features. In addition, Upsampling operation (red) and Concat operation (light green) were also used in the neck. The head consists of three Conv2d modules (yellow) and three detection heads (blue).

The neck network includes the CSP2, CBS, upsampling, and Concat operation. The CSP2 is composed of a concatenation of multiple CBS modules in two branches, which can further improve the ability of feature extraction. The composite structures of FPN and PAN are included in the neck network, thus producing a multi-scale fusion of features. The head network consists of three layers for object detection, which are used to output the prediction results of the target [29]. Figure 4 shows the YOLOv5s framework, which mainly consists of the backbone, neck, and head.

#### 2.2.2. Optimization of the Backbone Network

**Figure 5:**
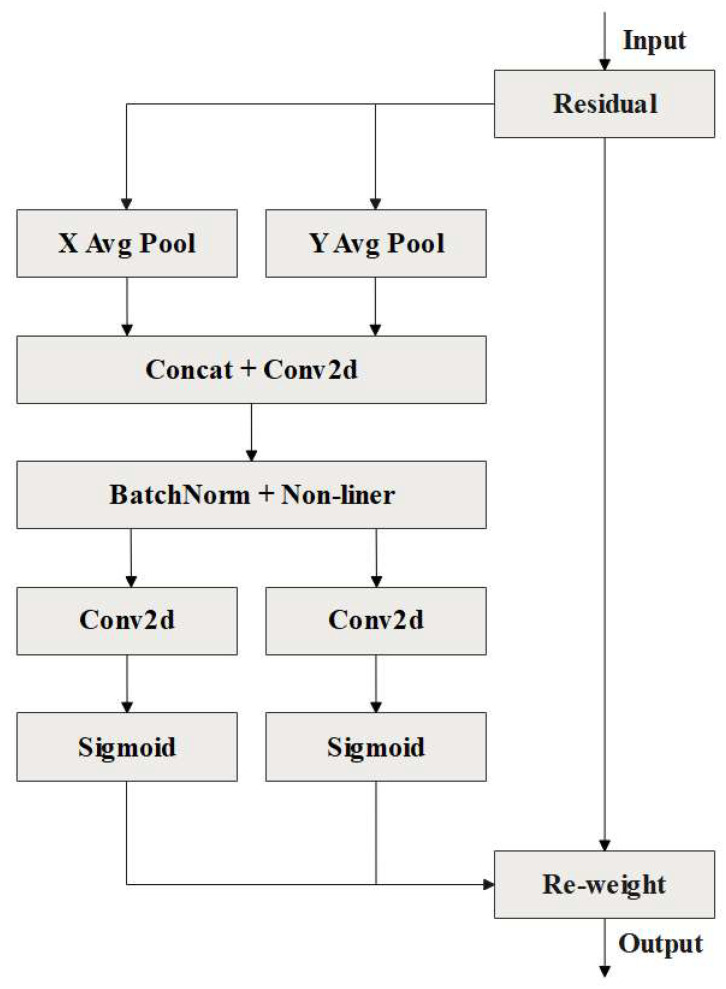
Structure diagram of the CA attention mechanism.

To further improve its feature extraction ability and efficiency in processing image information, the CA attention mechanism was introduced in the backbone. By embedding location information into channel attention, the CA attention mechanism enables the model to obtain information about a larger area. The CA attention mechanism contains both channel and spatial attention modules, outperforming SE with only the channel mechanism [30,31]. The structure diagram of the CA attention mechanism is shown in Figure 5.

The specific process of the CA module can be summarized as follows: $X={[x_{1},x_{2},\ldots ,x_{c}]\in R}^{C\times H\times W}$ denotes the input feature map, where C denotes the number of feature map channels, H denotes the height of the feature map, and W denotes the width of the feature map. The CA module first performs global average pooling on the input feature map in the height and width directions, and obtains feature maps of the height and width directions at the same time, which is shown as follows:

(1) $$\begin{array}{l} z_{c}^{h}\left(h\right)=\frac{1}{W}\sum_{0\leq i<W}x_{c}\left(h,i\right)\\ z_{c}^{w}\left(w\right)=\frac{1}{H}\sum_{0\leq j<H}x_{c}\left(j,w\right) \end{array}$$

where $z_{c}^{h}$ denotes the output of the c-th channel in a specific height direction, $z_{c}^{w}$ denotes the output of the c-th channel in a specific width direction, and $x_{c}$ denotes the input of the c-th channel.

The feature map obtained in Equation (1) is spliced and convolution transformed, and the dimensions of the feature map become C/r of the original, where r is the reduction factor. Then, after batch normalization and nonlinear activation, an intermediate feature map is obtained, as defined in Equation (2):

(2) $$f=\delta \left(F_{1}\left(\left[z^{h},z^{w}\right]\right)\right)$$

where f denotes the intermediate feature map obtained by encoding spatial information in the vertical and horizontal directions, δ denotes the nonlinear activation function, and $F_{1}$ denotes a 1 × 1 convolution transform.

The intermediate feature map f is divided into two independent tensors $f^{h}\in R^{C/r\times H}$ and $f^{w}\in R^{C/r\times W}$ along the spatial dimension, $f^{h}$ denotes the tensor decomposed along the height direction of the feature map f, and $f^{w}$ denotes the tensor decomposed along the width direction of the feature map f. Then, two 1 × 1 convolution transformations and the activation function δ are used to convert $f^{h}$ and $f^{w}$ into tensors with the same number of channels as the input feature map X. Finally, the attention weights on the height and width are obtained, as defined in Equation (3):

(3) $$\begin{array}{l} g^{h}=\sigma \left(F_{h}\left(f^{h}\right)\right)\\ g^{w}=\sigma \left(F_{w}\left(f^{w}\right)\right) \end{array}$$

where $g^{h}$ and $g^{w}$ denote weights in height and width, and σ is the sigmoid activation function. $F_{h}$ and $F_{w}$ denote convolution transformations in height and width.

The expanded attention weights $g^{h}$ and $g^{w}$ are multiplied with the input feature map X to obtain the output $Y=[y_{1},y_{2},\ldots ,y_{c}]$ of the CA module, as defined in Equation (4):

(4) $$y_{c}(i,j)=x_{c}(i,j)\times g_{c}^{h}(i)\times g_{c}^{w}(j)$$

where $y_{c}$ denotes the output of the c-th channel. $g_{c}^{h}$ and $g_{c}^{w}$ denote weights in height and width of the c-th channel.

**Figure 6:**
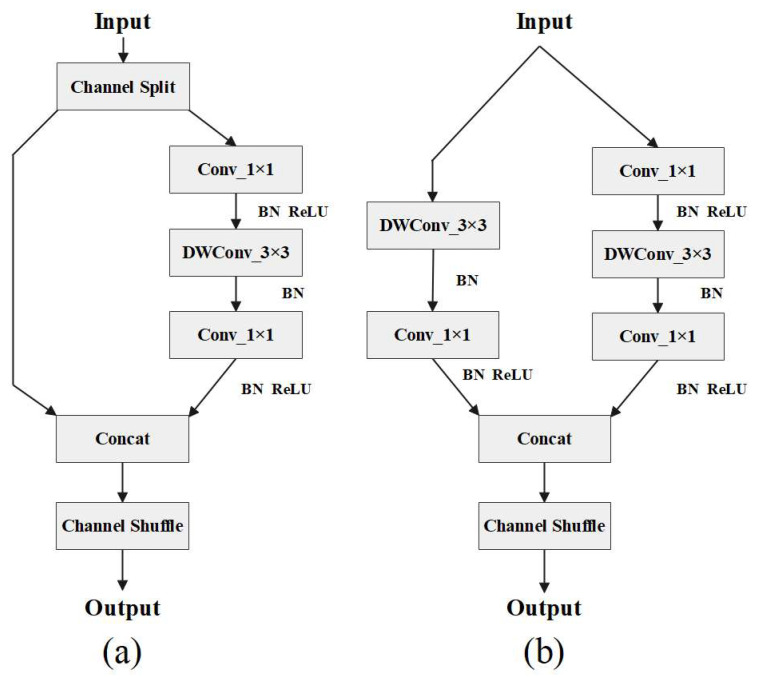
(**a**) is the network structure diagram of ShuffleNetv2 when the stride is 1, and (**b**) is the structure diagram of ShuffleNetv2 when the stride is 2.

ShuffleNetv2 is a lightweight network structure suitable for mobile terminals that has a good balance between speed and accuracy. ShuffleNetv2 is characterized by its maintenance of equal-width channels and non-use of intensive convolution operations to reduce memory access costs (MACs) and FLOPs of the model [32,33]. Figure 6 shows structure diagrams of ShuffleNetv2. When the stride is 1, ShuffleNetv2 conducts a channel-split operation, dividing the input feature map into two branches, with the number of channels being 1/2 each. The right branch passes through two ordinary convolutions and a depthwise separable convolution (DWConv). Then, the two branches conduct a Concat operation to fuse the features. A channel shuffle is used to exchange information between different groups so that channels are fully integrated. When the stride is 2, ShuffleNetv2 divides the feature map input into two branches. The left branch passes through an ordinary convolution and a DWConv. The right branch passes through two ordinary convolutions and a DWConv. Both branches use DWConv to reduce the dimensions of the height and width of the feature graph, thus reducing the FLOPs of the network. After the two branches are output, a Concat operation is performed to increase the network width. A channel shuffle is carried out to realize the information exchange between different channels [34].

**Figure 7:**
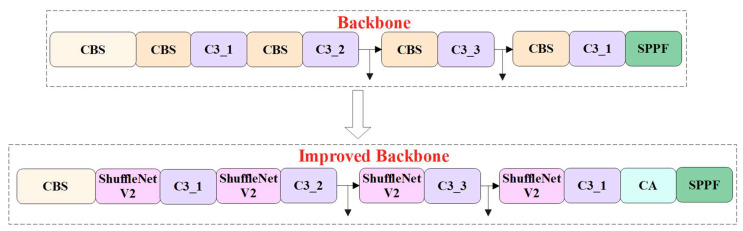
Schematic diagram of the optimized backbone network.

As shown in Figure 7, the CA module was introduced in front of the SPPF module to improve the ability of the improved model. Meanwhile, the ShuffleNetv2 block was used to replace the CBS module in the backbone of the YOLOv5s to ensure that the improved model does not lose too much accuracy based on its light weight.

#### 2.2.3. Optimization of the Neck Network

**Figure 8:**
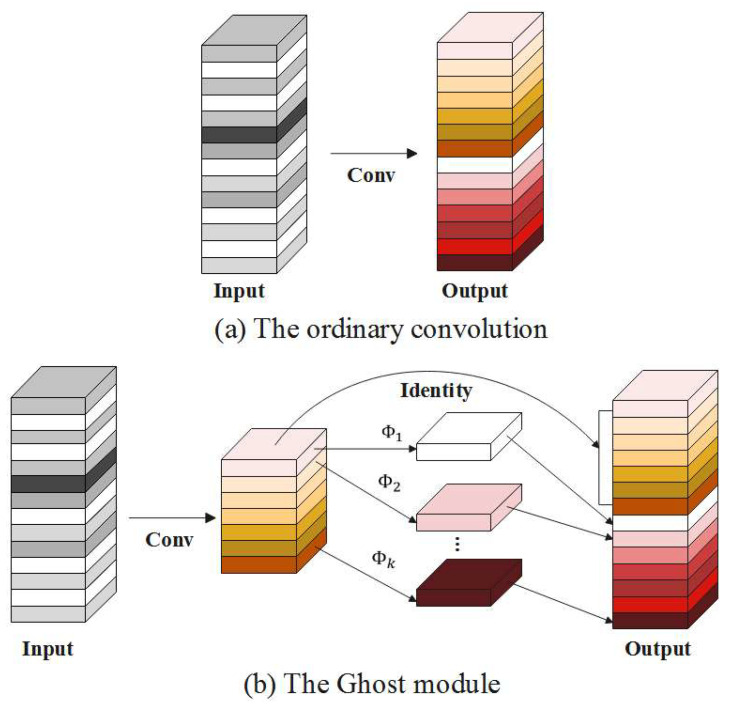
Schematic diagrams of ordinary convolution and the Ghost module. (**a**) is the operation flow of traditional convolution. Suppose that h×w×c is the size of the input feature map, and the convolution kernel is k×k, where c is the number of input channels, w is the width of input data, and h is the height of input data. The size of the output feature map is $h^{\prime }\times w^{\prime }\times n$, where $w^{\prime }$ and $h^{\prime }$ are the width and height of the output feature map, and n represents the number of output feature maps. The FLOPs of ordinary convolution can be calculated as $h^{\prime }\times w^{\prime }\times n\times c\times k\times k$. (**b**) is the Ghost module, which consists of two parts, including one part of ordinary convolution and the other part of the linear operation with less computation and fewer parameters. Through ordinary convolution, a total of m feature maps are obtained. After linear operation, a total of s new feature maps are generated from m feature maps, and the two sets of feature maps are spliced in a specified dimension. Finally, a total of n=m×s feature maps are generated in the Ghost module, and the size of the convolution kernel for each linear operation is d×d. The FLOPs of the Ghost module can be calculated as $h^{\prime }\times w^{\prime }\times m\times c\times k\times k+d\times d\times h^{\prime }\times w^{\prime }\times (n-m)$.

Although YOLOv5s has significant advantages in terms of parameters and FLOPs, we still find that it can be more lightweight to achieve satisfactory results. The Ghost module is a lightweight convolution structure that has fewer parameters and computations compared with traditional convolution structures [35,36]. The structure diagram of ordinary convolution is shown in Figure 8a, and the structure diagram of the Ghost module is shown in Figure 8b.

By comparing the FLOPs of the ordinary convolution with that of Ghost module, it can be found that the FLOPs ratio $r_{s}$ of the ordinary convolution relative to Ghost module is approximately equal to s, proving that the FLOPs of the Ghost module is smaller. The calculation process is defined in Equation (5).

(5) $$\begin{array}{ll} r_{S} & =\frac{w^{\prime }\cdot h^{\prime }\cdot n\cdot k\cdot k\cdot c}{w^{\prime }\cdot h^{\prime }\cdot m\cdot k\cdot k\cdot c+w^{\prime }\cdot h^{\prime }\cdot d\cdot d\cdot \left(n-m\right)}\\ & =\frac{w^{\prime }\cdot h^{\prime }\cdot n\cdot k\cdot k\cdot c}{w^{\prime }\cdot h^{\prime }\cdot \frac{n}{s}\cdot k\cdot k\cdot c+w^{\prime }\cdot h^{\prime }\cdot d\cdot d\cdot \left(s-1\right)\cdot \frac{n}{s}}\\ & =\frac{c\cdot k\cdot k}{c\cdot k\cdot k\cdot \frac{1}{s}+d\cdot d\cdot \frac{s-1}{s}}\approx \frac{s\cdot c}{s+c-1}\approx s \end{array}$$

where d×d has the similar size as that of k×k, and s≪c.

**Figure 9:**
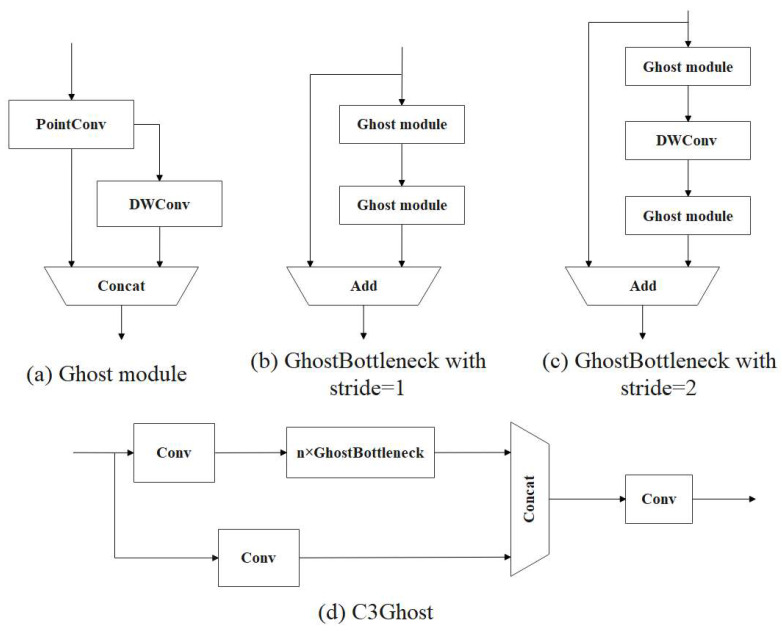
The specific structures of Ghost, GhostBottleneck, and C3Ghost. (**a**) is the structural diagram of Ghost module. The number of channels of the Ghost module is reduced to ½ of the number of output channels by 1 × 1 ordinary convolution. Then, a 5 × 5 DWConv is performed on the obtained feature map, and finally, the two sets of features are spliced. (**b**) is the structural diagram of the Ghost Bottleneck when the stride is 1. The Ghost Bottleneck consists of two stacked ghost modules. The first Ghost module is used for an extension layer that increases the number of channels. To match the number of channels for the input feature, the second Ghost module is used to reduce the number of channels. When the stride is 1, the two Ghost modules are directly performed, and then the input features and the output of the feature from the two Ghost modules are added for feature fusion. (**c**) is the structural diagram of the Ghost Bottleneck when the stride is 2. On the basis of the Ghost Bottleneck when the stride is 1, a DWConv with a step size of 2 is inserted between the two Ghost modules for downsampling. (**d**) is the structural diagram of C3Ghost module. The Bottleneck module in the C3 module is replaced with Ghost Bottleneck to form a C3Ghost structure. The new structure reduces the FLOPs and model size by replacing the 3 × 3 standard convolution in the original Bottleneck module.

Figure 9 shows the specific structures of the Ghost Bottleneck and C3Ghost models.

**Figure 10:**
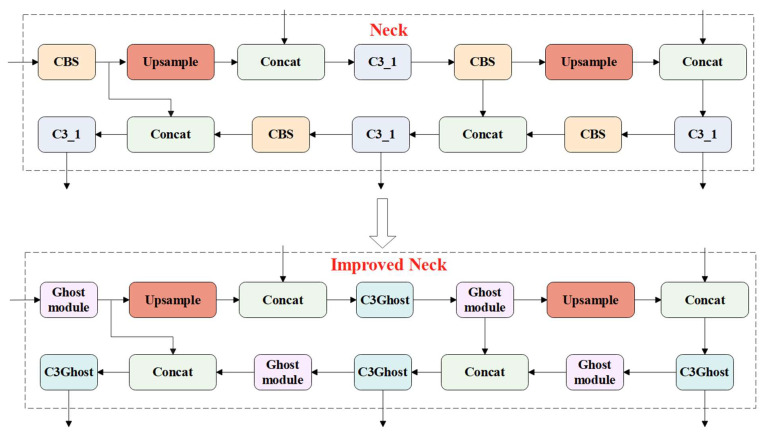
Schematic diagram of the improved neck network.

The ghost module is a lightweight convolution module [37,38]. To reduce the model size and FLOPs, the CBS module in the neck of the YOLOv5s model was replaced with the Ghost module, and the original C3 module was replaced with the C3Ghost module. The schematic diagram of the improved neck network is shown in Figure 10.

## 3. Results

### 3.1. Hyperparameters of Training

The experiment was conducted using a computer with the Windows 10 operating system, an i7-9700 (3.0 GHz) eight-core CPU, 16 G RAM, and NVIDIA RTX A5000 24 GB GPU. The hyperparameters were set as follows. The dynamic initialization learning rate was set to 0.001, the batch size was 16, and 50 training epochs were assigned for the training sets. The software platform was PyCharm, and the application software package was CUDA 11.3, PyTorch version 1.10.0, and Python version 3.8. All the training models used the same dataset and cross-validation method during the training process, calculating the average of multiple sets of training results as the final result.

### 3.2. Performance Evaluation

To evaluate the performance of LSR-YOLO, four evaluation metrics including average precision (AP), precision, recall, and mean average precision (mAP) were used. After predicting test samples, three states of precision and recall can be defined: true positive (TP), false positive (FP), and false negative (FN). Precision and recall are defined in Equations (6) and (7), respectively.

(6) $$\mathrm{Precision}=\frac{\mathrm{TP}}{\mathrm{TP}+\mathrm{FP}}$$

(7) $$\mathrm{Recall}=\frac{\mathrm{TP}}{\mathrm{TP}+\mathrm{FN}}$$

F1-scores can be used to evaluate the performance of models. The F1-score is defined in Equation (8).

(8) $$F1-\mathrm{score}=\frac{2\times \mathrm{Precision}\times \mathrm{Recall}}{\mathrm{Precision}+\mathrm{Recall}}$$

The definition of AP is given in Equation (9), and the definition of mAP is given in Equation (10):

(9) $$\mathrm{AP}=\int_{0}^{1}P(R)dR$$

(10) $$\mathrm{mAP}=\frac{\sum_{i=1}^{N}AP_{i}}{N}$$

where ${AP}_{i}$ denotes the average precision of target i, N denotes the total number of identified targets, P denotes the precision, R denotes the recall, and mAP@0.5 denotes the average AP of all categories when IOU is set to 0.5.

The average detection time refers to the average time taken by the trained model to recognize each sheep face image. Specifically, there were ten test images in total, each group of models performed ten tests on the test images, and the average detection time was calculated after the test. Model size refers to the weight of the model saved after final training.

### 3.3. Training Evaluation

**Figure 11:**
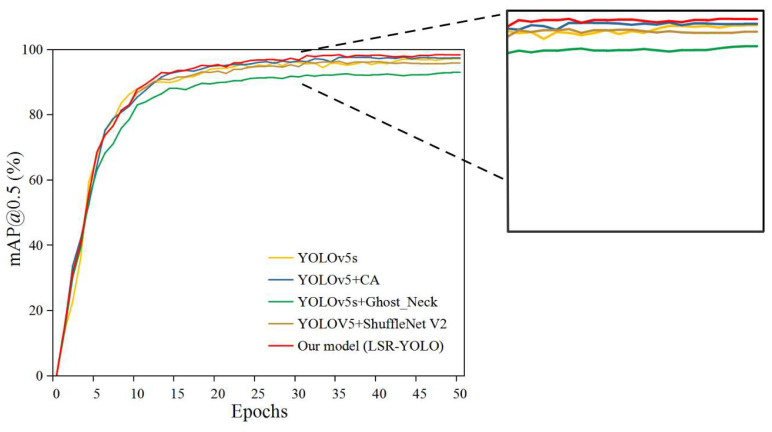
Training curves of multiple groups of improved models.

To evaluate the performance of LSR-YOLO, five types of models were configured: YOLOv5s, YOLOv5s + CA, YOLOv5s + ShuffleNetv2, YOLOv5s + Ghost_Neck, and LSR-YOLO. Based on the YOLOv5s model, we further established YOLOv5s + CA, YOLOv5s + ShuffleNetv2, and YOLOv5s + Ghost_Neck by introducing the improvement strategies used in this study separately, to further explore the effects of introducing improved modules. The variation curves of mAP@0.5 are shown in Figure 11. During the initial stage of training, the value of mAP@0.5 increased rapidly. When the number of training epochs reached 45, the curves gradually stabilized. The LSR-YOLO model achieved the best performance in the sheep face dataset, with a stable mAP@0.5 at 97.8%. In addition, through the curve, we found that compared to YOLOv5s, the introduction of the CA module improved the mAP@0.5, proving that the introduction of the attention mechanism can improve the performance of the model. Compared to YOLOv5s, after introducing the ShuffleNetv2 module and Ghost module, the recognition performance of the improved model decreased, proving that the lightweight module would cause a loss in model performance. The curve proves that the LSR-YOLO model can effectively learn target characteristics and achieve better training effects for the sheep face dataset of this study.

### 3.4. Comparison with Different Detection Models

Several sets of classical target detection models—YOLOv3-tiny, YOLOv4-tiny, VGG16, SSD, and YOLOv5s—were used to train the sheep face dataset. The training results are shown in Table 2. As shown in Table 2, YOLOv5s achieved the best performance on the sheep face dataset, in which the precision rate reached 93.4%, the recall rate reached 95.4%, and the F1-score reached 94.4%. Compared to the models listed above, the F1-score of YOLOv5s is 11.8%, 7.7%, 9.9%, and 2.3% higher, respectively. In addition, the model size of YOLOv5s is only 14.0 MB, which has significant advantages in being deployed on mobile devices. YOLOv3-tiny and YOLOv4-tiny are lightweight models proposed in the YOLO series, but they are inferior to YOLOv5s in terms of recognition accuracy and model size. The model sizes of VGG16 and SSD are relatively large, so they are not suitable for mobile terminal recognition in this study. In contrast, YOLOv5s has significant advantages in model accuracy and model size. Therefore, YOLOv5s was selected as the basic model in this study, and various targeted improvement strategies will be carried out in the future.

### 3.5. Improved Module Performance Comparison

To evaluate the specific performance of the improved modules, we used YOLOv5s as a benchmark model to verify model performance by adding different modules. The experimental results are shown in Table 3. Specifically, compared with the YOLOv5s model, the replacement of C3Ghost and Ghost modules in the neck of the model reduced the number of parameters by 1,403,536, the FLOPs by 2.5 G, the average detection time by 1.4 ms, and the model size by 2.7 MB. Meanwhile, the mAP@0.5 was reduced by 4.0%. The comparison results show that the replacement of C3Ghost and Ghost modules in the neck of the model reduced the model size, FLOPs, and average detection time but also reduced the performance of the model. Compared with the YOLOv5s model, after introducing the CA module to the backbone part, the mAP@0.5 increased by 0.7%. The experiment shows that the performance of the model can be improved after the introduction of the CA module with slightly increased model parameters. Compared with the YOLOv5s model, replacing the CBS module in the backbone with the ShuffleNetv2 module further reduced the model parameters, FLOPs, average detection time, and model size by 1,294,080, 2.4 G, 1.5 ms, and 2.4 MB, respectively. The introduction of the ShuffleNetv2 module reduced the mAP@0.5 by 0.9%. After the introduction of the Ghost module and ShuffleNetv2 module, the model size of the improved model was only 9.0 MB, and the single-image recognition speed reached 9.0 ms, the best performance in terms of model volume and recognition speed. However, the mAP@0.5 of the improved model was only 93.9%. Compared with YOLOv5s, the mAP@0.5 was reduced by 3.9%, so it was not suitable for the sheep face recognition task in this study. From the table, LSR-YOLO achieved good performance in the sheep face dataset. Compared to YOLOv5s, the model size of LSR-YOLO was reduced by 4.5 MB and the mAP@0.5 was increased by 0.8%. In conclusion, the LSR-YOLO model achieves a good balance in FLOPs, model size, and detection accuracy.

**Figure 12:**
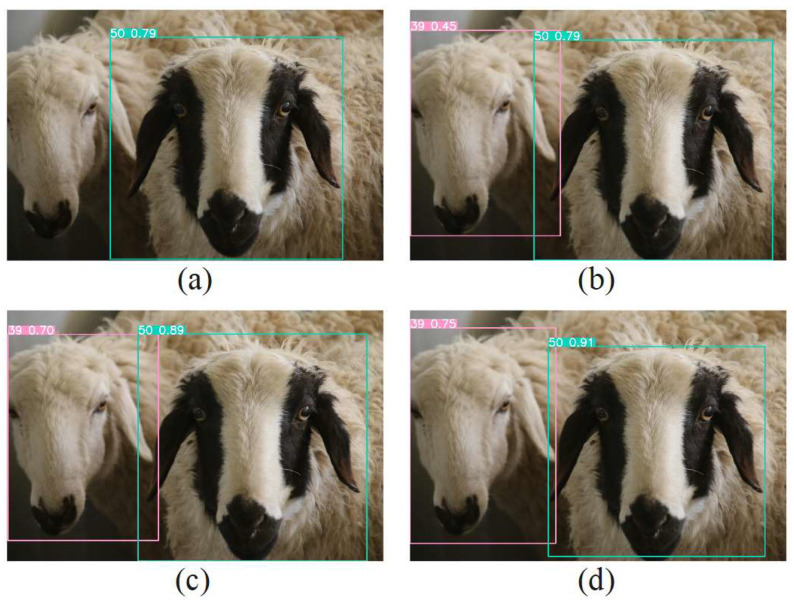
Recognition results of different improved models, including (**a**) the YOLOv5s + Ghost_Neck model, (**b**) the YOLOv5s model, (**c**) the YOLOv5s + CA model, and (**d**) the LSR-YOLO model. For different recognition targets, the model marks recognition boxes with different colors, and the colors of the recognition boxes are random.

To test the recognition effect of the improved model, a sample image was randomly selected and different improved models were used for recognition. The recognition results are shown in Figure 12. Figure 12a shows the recognition effects of the YOLOv5s + Ghost_Neck model, Figure 12b shows the recognition effects of the YOLOv5s model, Figure 12c shows the recognition effects of the YOLOv5s + CA model, and Figure 12d shows the recognition effects of the LSR-YOLO model. Among them, LSR-YOLO achieved the best performance and the highest confidence degree in the recognition task.

### 3.6. Improved Backbone Performance Comparison

In this study, we proposed several improvement schemes for the backbone of YOLOv5s and further discussed their specific effects. Specifically, we replaced the CBS module in the backbone with the RepVGG module and ShuffleNetv2 module. The experimental results are shown in Table 4. From the results in the table, the introduction of ShuffleNetv2 achieved lightweight improvements without losing too much recognition accuracy. Compared with YOLOv5s, the number of parameters of YOLOv5s + ShuffleNetv2 decreased by 1,294,080, while single image recognition time decreased by 1.5 ms and model size decreased by 2.4 MB. Meanwhile, after introducing the ShuffleNetv2 module, the mAP@0.5 of the improved model was reduced by 0.9%. Compared with YOLOv5s, YOLOv5s + RepVGG increased the parameters by 176,000, the average detection time by 0.1 ms, and the model size by 0.3 MB and reduced the mAP@0.5 by 0.1%. The experimental results indicate that the effects of introducing the RepVGG module were poor. In summary, the introduction of ShuffleNetv2 can achieve lightweight improvement without losing too much accuracy.

### 3.7. Ghost Module Performance Comparison

To explore the effects of introducing the Ghost and C3Ghost modules into the YOLOv5s model, we replaced the common convolution and C3 structure of the backbone, neck, and all parts of YOLOv5s. As shown in Table 5, a total of five groups of models were compared after training, including YOLOv5s, YOLOv5s + Ghost_Backbone, YOLOv5s + Ghost_all, YOLOv5s + Ghost_Neck, and LSR-YOLO. In the YOLOv5s + Ghost_all model, the ordinary convolution and C3 structures in the backbone and neck were replaced by lightweight modules. The YOLOv5s + Ghost_Backbone model replaced all the ordinary convolution and C3 structures of the backbone part with lightweight modules. Compared with the YOLOv5s model, after introducing the C3Ghost and Ghost modules in the backbone, the parameters, FLOPs, average detection time, model size, and mAP@0.5 were reduced by 1,934,248, 5.2 G, 1.7 ms, 3.7 MB, and 6.1%, respectively. The experimental results show that YOLOv5s + Ghost_Backbone lost a significant amount of recognition accuracy. As a result of the large number of convolutions in the backbone of YOLOv5s, the introduction of the C3Ghost module and Ghost module greatly reduced the feature extraction ability of the improved model.

Compared to YOLOv5s, after introducing the C3Ghost and Ghost modules in the neck of YOLOv5s, the parameters, FLOPs, average detection time, model size, and mAP@0.5 were reduced by 1,403,536, 2.5 G, 1.4 ms, 2.7 MB and 4.0%, respectively. When the C3Ghost and Ghost modules were introduced in both the backbone and neck of YOLOv5s, the parameters, FLOPs, average detection time, model size, and mAP@0.5 were reduced by 3,337,784, 7.8 G, 4.3 ms, 6.3 MB and 14.6%, respectively. Although YOLOv5s + Ghost_all achieved the best results in terms of detection speed and model size, the recognition accuracy loss of the model was too great, so it was not suitable for sheep face recognition tasks.

The above results show that when the Ghost and C3Ghost modules are introduced into the neck network, the improved model had the smallest loss based on lightweight improvement. Therefore, we introduced the C3Ghost and Ghost modules into the neck part to achieve the effects of lightweight improvement.

### 3.8. Comparison of Different Attention Modules

The effects of introducing CA were further determined by introducing different attention mechanism modules. The specific experiments were performed as follows: other attention mechanism modules, such as SE (Sequence and Exception module), ECA (Effective Channel Attention module), and CBAM (Convolutional Block Attention Module), were embedded in the front of the SPPF module of the YOLOv5 + ShuffleNetv2 + Ghost_Neck model. The experimental results are shown in Table 6.

From Table 6, it can be seen that after embedding the CA module, the mAP@0.5 of the improved model reached 97.8%, which is the highest recognition accuracy achieved. Compared to the introduction of the other three attention mechanism modules, the mAP@0.5 increased by 0.5%, 0.6%, and 0.2%. In addition, after introducing different attention mechanisms, the model size of the improved model was similar, ranging from 9.4 MB to 9.5 MB. The improvement in detection accuracy brought by the introduction of the CA module was higher than that of other attention mechanism modules. Therefore, the CA attention mechanism was introduced to improve the performance of the improved model.

### 3.9. Comparison with State-of-the-Art Models

To explore the performance of the LSR-YOLO proposed in this study, we compared it with the sheep face recognition models proposed in previous studies. Previous studies have used models including YOLOv3, Resnet50, and YOLOv4 [15,16,17]. The comparison results are shown in Table 7. As can be seen from Table 7, LSR-YOLO achieved the highest F1-score, which was 3.9%, 1.9%, and 0.9% higher than the other three groups of models. In addition, compared to the models proposed in previous studies, the model size of LSR-YOLO was only 9.5 MB, which is more friendly for mobile devices. The comparison results show that LSR-YOLO has significant advantages in model performance and application prospects.

## 4. Discussion

In this study, a lightweight sheep face recognition model named LSR-YOLO was constructed to recognize the corresponding identity of a sheep face image. LSR-YOLO has great advantages in recognition accuracy and model size. The experimental results on the self-made sheep face dataset show that the mAP@0.5 of the LSR-YOLO model reaches 97.8%. In addition, the model size of LSR-YOLO is only 9.5 MB, which provides a method for the deployment of a mobile terminal identification system.

The sheep face dataset in this study only collected facial images of small-tailed Han sheep, and there may be deviations in the recognition results of other breeds of sheep. Therefore, in future research, we will continue to expand the scale of the sheep face dataset by adding facial images of more breeds of sheep to further increase the diversity of the dataset.

**Figure 13:**
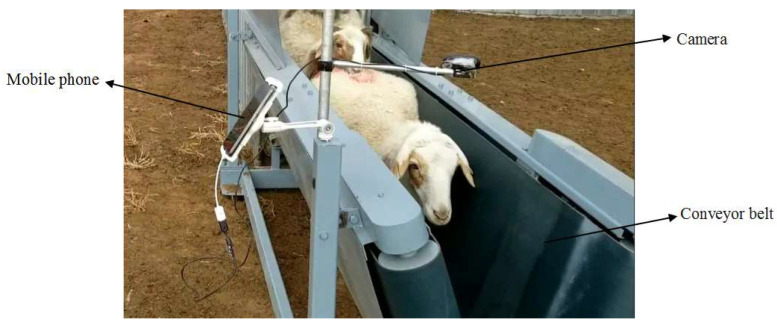
The sheep facial image acquisition device.

Sheep face image acquisition is difficult because sheep are emotionally sensitive and prone to extreme behavior. Therefore, it will be beneficial to develop a sheep face image acquisition device to solve these problems. The acquisition device can be paired with a server system to save the acquired images, and the model can be retrained on the face images of newly arrived sheep. This method would be expected to identify more sheep. To solve the above problems, we designed a set of sheep facial image acquisition devices, their structure mainly including a mobile phone, camera, and conveyor belt. The sheep facial image acquisition device is shown in Figure 13. Specifically, the mobile phone is connected to a USB camera to video-record the sheep’s facial images passing through the conveyor belt. The two groups of conveyor belts form a V-shaped structure to fix the body of the sheep. When the sheep pass through the conveyor belt structure, the conveyor belt helps the sheep move forward to prevent the sheep from stopping and causing congestion. In addition, the conveyor belt structure can prevent sheep from having a stress reaction. At present, the equipment is in the testing stage, and we will promote it in the future.

Face images of individuals from the same breed of sheep are highly similar. Taking the small-tailed Han sheep as an example, some sheep have different details, including yellow spots, black spots, and ear shapes. By collecting sheep face images from multiple perspectives for training, the recognition model can learn richer and more robust details, thus improving its recognition accuracy. In the future, we will optimize the sheep facial image acquisition device to achieve the effects of collecting multiple facial images at the same time.

In the long run, developing a mobile recognition system—specifically, integrating a lightweight sheep face recognition model into a mobile phone and developing a sheep face recognition application—would be beneficial. Herders could access real-time information about sheep through their mobile phones, further increasing management efficiency. In addition, herdsmen could also use the camera on their mobile phones to capture target images, further identifying target identities and obtaining target information. Mobile end recognition would provide herdsmen with a more convenient and efficient recognition method. The identified results could be transmitted to a centralized server on the farm through a local area network on a 5G network. Our vision is to propose a lightweight sheep face recognition model for sheep face recognition, thereby reducing recognition time and saving deployment costs.

**Figure 14:**
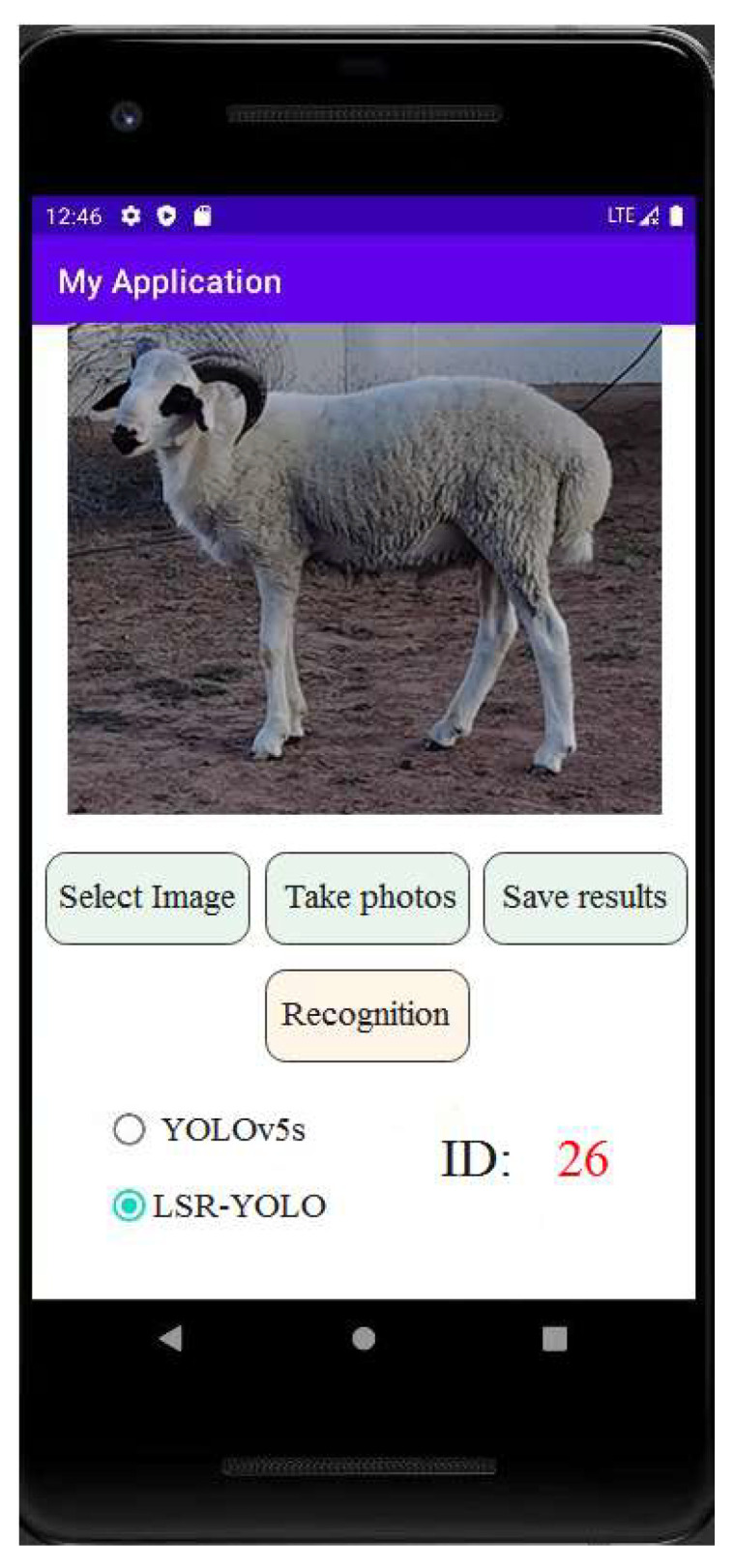
Interface of the mobile sheep face recognition system.

In this study, we developed a mobile sheep face recognition system. This recognition system was designed using Android Studio. The sheep face recognition system is divided into three modules: image selection, image display, and recognition results. Each module was designed independently to realize the functions of image recognition, analysis, and preservation. A sheep face recognition control system can recognize sheep face images effectively and provide corresponding identity information in the system. Administrators can view, update, and save information in real-time. The interface of the mobile sheep face recognition system is shown in Figure 14.

In future research, more models in the field of computer vision need to be evaluated and developed to face the challenge of sheep face recognition. In long-term planning, sheep face recognition is also meant to develop models for livestock tracking, counting, emotional analysis, and weight estimation. By integrating multiple algorithms, the collected information is transmitted in real-time to the central server of the farm, achieving the construction of big data farms and meeting the needs of modern and welfare farming [39,40]. The sheep face dataset used in this study currently has project partnerships with some companies, and as the project collaboration has not yet ended, the dataset is currently not publicly available. In the future, we will consider making this dataset publicly available for easy access.

## 5. Conclusions

In this study, we applied deep learning technology to sheep face recognition detection and proposed an improved, lightweight sheep face detection model based on YOLOv5s. Lightweight modules, including the ShuffleNetv2, Ghost, and C3Ghost modules, were introduced into YOLOv5s to reduce its model size and FLOPs. The CA attention module was introduced into the backbone to select critical information and suppress uncritical information, thereby improving the performance of the model.

From the results, the LSR-YOLO model achieved the best recognition results, and the mAP@0.5 of the sheep face dataset reached 97.8%. Through a more animal welfare-friendly identification method, the harm caused by traditional identification methods to individual animals can be avoided. The model size of the LSR-YOLO is only 9.5 MB, and the research results provide technical support for the development of mobile sheep face recognition system.

## Figures and Tables

**Table 1:** Specifications of the sheep face dataset.

Dataset	Images	Size	Proportion
Training	9928	2736 × 1824	80%
Verification	1241	2736 × 1824	10%
Testing	1241	2736 × 1824	10%
Total	12,410	2736 × 1824	100%

**Table 2:** Results of different detection models.

Model	Precision (%)	Recall (%)	F1-Score (%)	Model Size (MB)
YOLOv3-tiny	82.0	83.2	82.6	33.7
YOLOv4-tiny	86.0	87.5	86.7	22.6
VGG16	86.2	82.8	84.5	527.8
SSD	91.3	93.0	92.1	99.5
YOLOv5s	93.4	95.4	94.4	14.0

**Table 3:** Results of introducing different improvement modules. The “√” in the table represents the use of the improved module and model.

YOLOv5s	Ghost_Neck	ShuffleNev2	CA	Parameters	Average Detection Time (ms per Image)	FLOPs (G)	Model Size (MB)	mAP@0.5 (%)
√				7,189,540	12.5	16.5	14.0	97.0
√	√			5,786,004	11.1	14.0	11.3	93.0
√		√		5,895,460	11.0	14.1	11.6	96.1
√			√	7,483,476	12.6	17.2	14.5	97.7
√	√	√		4,491,924	9.0	11.6	9.0	93.9
√	√		√	6,079,940	11.6	14.7	11.9	94.8
√		√	√	6,189,396	10.2	14.8	12.1	96.8
√	√	√	√	4,785,860	9.3	12.3	9.5	97.8

**Table 4:** Results of introducing different modules into the backbone network.

Model	Parameters	FLOPs (G)	Average Detection Time (ms per Image)	Model Size (MB)	mAP@0.5 (%)
YOLOv5s	7,189,540	16.5	12.5	14.0	97.0
YOLOv5s + RepVGG	7,365,540	16.9	12.6	14.3	96.9
YOLOv5s + ShuffleNetv2	5,895,460	14.1	11.0	11.6	96.1

**Table 5:** Results of introducing Ghost modules at different locations.

Model	Parameters	FLOPs (G)	Average Detection Time (ms per Image)	Model Size (MB)	mAP@0.5 (%)
YOLOv5s	7,189,540	16.5	12.5	14.0	97.0
YOLOv5s + Ghost_all	3,851,756	8.7	8.2	7.7	82.4
YOLOv5s + Ghost_Backbone	5,255,292	11.3	10.8	10.3	90.9
YOLOv5s + Ghost_Neck	5,786,004	14.0	11.1	11.3	93.0

**Table 6:** Results of introducing different attention mechanisms.

Group	Model	mAP@0.5 (%)	Model Size (MB)
1	+ECA	97.3	9.4
2	+SE	97.2	9.5
3	+CBAM	97.6	9.5
4	+CA (ours)	97.8	9.5

**Table 7:** Comparison of the research results of other sheep face recognition.

Model	Precision (%)	Recall (%)	F1-Score (%)	Model Size (MB)
Song et al. (2022) [15]	89.9	97.5	93.5	61.5
Billah et al. (2022) [16]	96.0	95.0	95.5	244.3
Hitelman et al. (2022) [17]	97.0	96.0	96.5	98.1
Our study	97.1	97.6	97.4	9.5

## Data Availability

Not applicable.
